# Long non-coding RNA profile study identifies a metabolism-related signature for colorectal cancer

**DOI:** 10.1186/s10020-021-00343-x

**Published:** 2021-08-03

**Authors:** Yongqu Lu, Wendong Wang, Zhenzhen Liu, Junren Ma, Xin Zhou, Wei Fu

**Affiliations:** Department of General Surgery, Peking University Third Hospital, 49 North Garden Road, Haidian District, Beijing, 100191 China

**Keywords:** Bioinformatics, Colorectal cancer, Long non-coding RNA, Metabolism-related gene, Prediction model, Risk score

## Abstract

**Background:**

Heterogeneity in colorectal cancer (CRC) patients provides novel strategies in clinical decision-making. Identifying distinctive subgroups in patients can improve the screening of CRC and reduce the cost of tests. Metabolism-related long non-coding RNA (lncRNA) can help detection of tumorigenesis and development for CRC patients.

**Methods:**

RNA sequencing and clinical data of CRC patients which extracted and integrated from public databases including The Cancer Genome Atlas (TCGA) and Gene Expression Omnibus (GEO) were set as training cohort and validation cohort. Metabolism-related genes were acquired from Kyoto Encyclopedia of Genes and Genomes (KEGG) and the metabolism-related lncRNAs were filtered using correlation analysis. The risk score was calculated based on lncRNAs with prognostic value and verified through survival curve, receiver operating characteristic (ROC) curve and risk curve. Prognostic factors of CRC patients were also analyzed. Nomogram was constructed based on the results of cox regression analyses. The different immune status was observed in the single sample Gene Set Enrichment Analysis (ssGSEA).

**Results:**

The training cohort and the validation cohort enrolled 432 and 547 CRC patients respectively. A total of 23 metabolism-related lncRNAs with prognostic value were screened out and 10 of which were significantly differentially expressed between tumour and normal tissues. Finally, 8 lncRNAs were used to establish a risk score (DICER1-AS1, PCAT6, GAS5, PRR7-AS1, MCM3AP-AS1, GAS6-AS1, LINC01082 and ADIRF-AS1). Patients were divided into high-risk and low-risk groups according to the median of risk scores in training cohort and the survival curves indicated that the survival prognosis was significantly different. The area under curve (AUC) of the ROC curve in two cohorts were both greater than 0.6. The age, tumour stage and risk score were selected as independent factors and used to construct a nomogram to predict CRC patients' survival rate with the c-index of 0.806. The ssGSEA indicated that the risk score was associated with immune cells and functions.

**Conclusions:**

Our systematic study established a metabolism-related lncRNA signature to predict outcomes of CRC patients which may contribute to individual prevention and treatment.

**Supplementary Information:**

The online version contains supplementary material available at 10.1186/s10020-021-00343-x.

## Introduction

Colorectal cancer (CRC), a common malignant tumour in the digestive system, ranks third in terms of incidence and second in mortality according to global cancer statistics (Bray et al. 2018). Patients with early-stage CRC rarely have obvious symptoms, while an increase in relational discomfort such as abdominal pain and haematochezia may indicate tumour progression. There is a striking link between tumour development and the prognosis of patients, and this link directly affects therapeutic options and outcomes (Dekker et al. 2019). Therefore, the detection of CRC at the earliest possible period is of paramount importance for treatment. The indicators commonly used in the clinic to predict the prognosis and assess the risk factors of CRC include the pathological assessment of the resected specimen and serological tests, such as carcinoembryonic antigen (CEA) assessment (Labianca et al. 2013). With the improvement in high-throughput genome sequencing technologies, genetic information is applied for broader usage, and comprehensive analysis can reflect the biological characteristics of tumorigenesis and progression for individuals (Bian et al. 2018; Carethers and Jung 2015).

The status of cell proliferation in tumour progression involves the corresponding alterations in cellular metabolism (Hanahan and Weinberg 2011). The features of metabolism in tumours, such as the Warburg effect, significantly differ from the processes in normal tissues as a result of adaptative reprogramming (Vander Heiden et al. 2009). Alterations in the activities and contents of metabolites may effectively fuel tumour growth (Jones and Thompson 2009). As the metabolic activities between proliferating cells and nonproliferating cells are fundamentally different, the screening of metabolic biomarkers can specifically detect abnormal changes in organisms for the prevention of malignant diseases with pathophysiological characteristics (DeBerardinis et al. 2008). Related studies that focus on metabolism also provide new ideas for the development of new drugs and diagnostic methods. High-throughput analytical technology reveals the metabolic changes in body fluid and tissues that are potentially associated with carcinogenesis mechanisms of CRC (Ni et al. 2014). Metabolomics has been confirmed to be advantageous in CRC biomarker discovery for early diagnosis and prognosis and has advantages over conventional strategies in terms of sensitivity and specificity (Zhang et al. 2014).

In this study, we aimed to identify a metabolism-related signature of CRC patients based on a profile study of long non-coding RNAs (lncRNAs). We obtained the transcriptome data of CRC patients from public databases and screened out lncRNAs correlated with metabolism-related genes with significant clinical value. Using these lncRNAs, we developed a prognostic scoring system and verified the accuracy with an external cohort. Additionally, the expression features of included lncRNAs were verified in immune microenvironment. A novel model combining the metabolic risk score and clinical parameters was constructed and was able to predict the prognosis of CRC patients.

## Methods

### Data extraction

The expression profiles, including RNA sequencing data, and the corresponding clinical data of CRC patients were downloaded from The Cancer Genome Atlas (TCGA, https://portal.gdc.cancer.gov) and the Gene Expression Omnibus (GEO microarray dataset GSE39582 based on the GPL570 platform, https://www.ncbi.nlm.nih.gov/geo) database (Edgar et al. 2002; Hutter and Zenklusen 2018). The enrolled patients had a definite diagnosis of CRC, and their overall survival (OS) time was not less than 30 days. Patients without available data for age, sex and pathologic stage (tumour-node-metastasis, TNM) were excluded. We performed calibrations and log2 transformations with the sva package for batch normalization. Finally, 432 CRC patients from the TCGA were used as the training cohort, and 547 patients from the GEO were used as the validation cohort (Additional file 1: Table S1).

### Identification of metabolism-related lncRNAs

We identified metabolism-related genes based on Kyoto Encyclopedia of Genes and Genomes (KEGG, https://www.gsea-msigdb.org/gsea/msigdb) gene sets from the Molecular Signatures Database, which contains metabolism-related pathways (Kanehisa and Goto 2000). Coefficients were calculated to determine the correlation between the metabolism-related genes and the expression of corresponding lncRNAs. The metabolism-related lncRNAs with an absolute value of the correlation coefficient greater than 0.4 and the P-value less than 0.05 were selected.

### Construction of the prognostic signature

The differentially expressed metabolism-related lncRNAs between tumour and normal tissues in the training cohort were selected with the limma package with cut-offs of fold change (FC) ≥ 2 and false discovery rate (FDR) ≤ 0.05. Metabolism-related lncRNAs whose expression levels were significantly associated with the OS of the training cohort were screened out, and hazard ratios (HRs) were used to identify risk factors (HR > 1) and protective factors (HR < 1). We intersected the two lncRNA sets as candidate metabolism-related lncRNAs and subjected them to analysis to evaluate their contribution as independent prognostic factors in CRC patients. The corresponding coefficients for different metabolism-related lncRNAs in the model were confirmed after statistical estimation with the glmnet package. A risk score formula was constructed to predict patient prognosis: risk score = Σ coefficient of lncRNA i * expression value of lncRNA i.

### Validation of the risk score

On the basis of the median value of the risk scores in the training cohort, the patients in the two cohorts were divided into two groups: the high-risk group and the low-risk group. The predictive value of the risk score was assessed by survival curve, risk curve and receiver operating characteristic (ROC) curve analysis with the survival and survivalROC packages. Principal component analysis (PCA) was performed to visualize the lncRNA expression patterns in the CRC patients in different groups.

### Clinical parameter correlation analysis

Correlation analysis between the risk score and the clinical parameters of the training cohort was performed to explore the association of the prognostic signature with other characteristics.

### Evaluation of the prognostic signature

The mRNA-lncRNA co-expression network was constructed, and the correlations between the metabolism-related lncRNAs and their target mRNAs were visualized by Cytoscape (version 3.7.1). The corrplot package was used to analyse interactions between selected lncRNAs. The co-expressed network components were depicted with a Sankey diagram.

Functional enrichment analyses, including gene ontology (GO) analysis and KEGG analysis, were conducted to investigate the biological functions and pathways related to the selected lncRNAs.

The enrichment levels of immune signatures featuring associated markers were analysed by single-sample gene set enrichment analysis (ssGSEA). Gene markers of immune signatures, including antigen presenting cell (APC) co-inhibition, APC co-stimulation, chemokine receptors (CCR), check point, cytolytic activity, human lymphocyte histocompatibility antigen (HLA), inflammation promoting, major histocompatibility complex (MHC) class I, parainflammation, T cell co-inhibition, T cell co-stimulation, Type I interferon (IFN) response, Type II IFN response, dendritic cells (DCs), activated DCs (aDCs), B cells, CD8^+^ T cells, immature DCs (iDCs), macrophages, mast cells, neutrophils, natural killer (NK) cells, plasmacytoid DCs (pDCs), T helper (Th) cells, T follicular helper (TFH) cells, Th1 cells, Th2 cells, tumour-infiltrating lymphocytes (TILs) and regulatory T cells (Tregs), were obtained from previous studies (Bindea et al. 2013; Charoentong et al. 2017). The correlations of the metabolic risk score with immune infiltration levels were also analysed by assessing the infiltration data of CRC patients from the Tumour Immune Estimation Resource (TIMER) (Li et al. 2017).

### Construction of the nomogram

The risk score and clinical parameters were analysed to screen out independent risk factors in CRC patients from the training cohort. Based on the identified variables, a nomogram was constructed for predicting one-, three- and five-year OS, the prognostic value of the nomogram was visualized with the rms package. The concordance index (C-index) was calculated to evaluate the predictive ability of the nomogram. Calibration curves were depicted to verify the concordance between predicted survival and observed survival after bias correction.

### Statistical analysis

All statistical analyses were performed in R (version 3.6.0). Pearson test was conducted for correlation analysis. The Wilcoxon test and Kruskal-Wallis test were used in differential analyses. Univariate cox proportional hazards regression was used to estimate the HRs. Coefficients of the prognostic signature were calculated by least absolute shrinkage and selection operator (LASSO) regression. The survival curve was generated by the Kaplan-Meier method. OS and relapse free survival (RFS) differences were evaluated using the log-rank test. Pearson test was conducted for correlation analysis. We obtained independent risk factors for the prognosis of CRC patients by univariate cox analysis and multivariate cox analysis. The confidence interval (CI) was set at 95%, and a P-value < 0.05 was considered to indicate a significant difference in the statistical analyses.

## Results

### Screening of metabolism-related lncRNAs

**Fig. 1:**
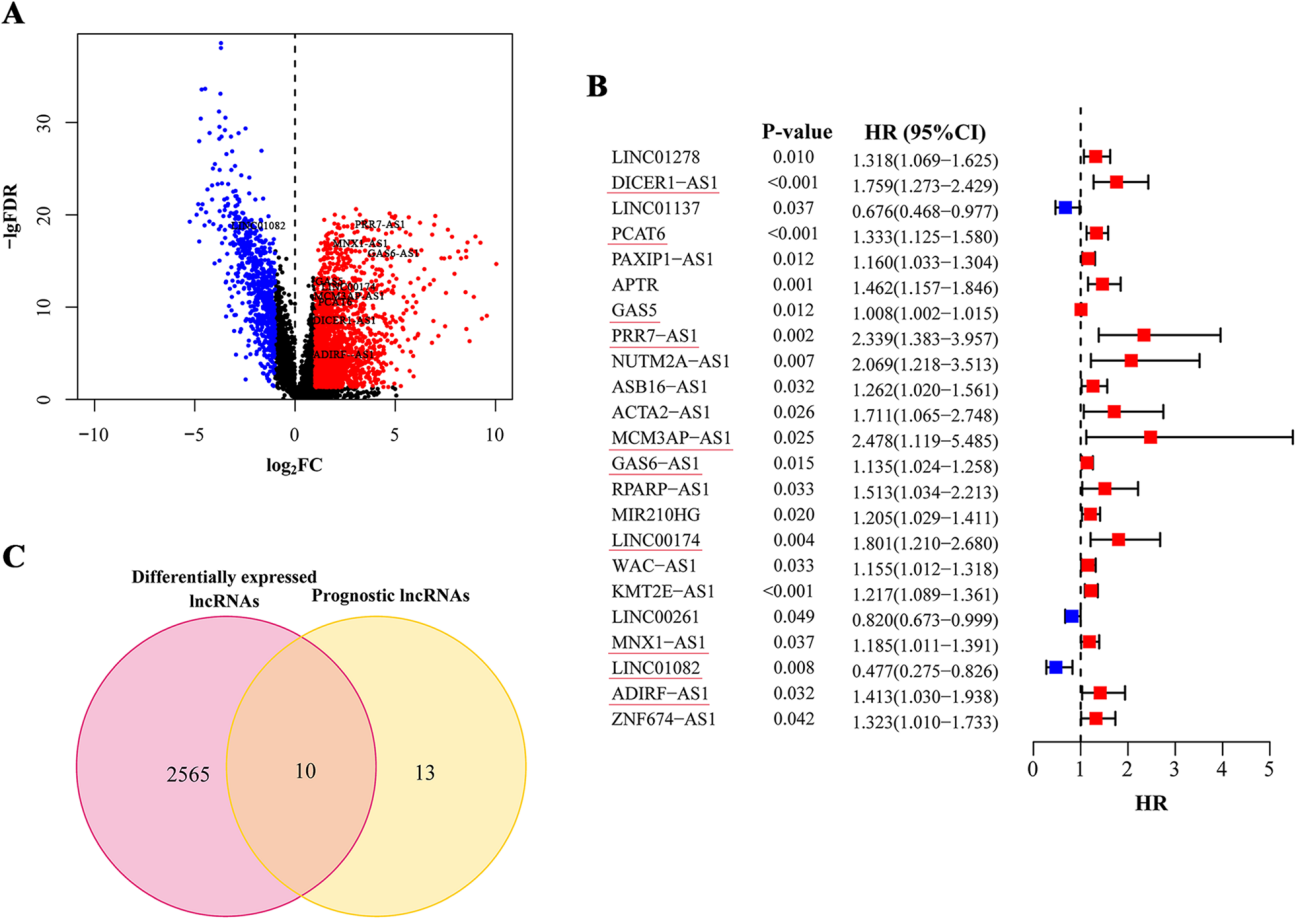
Filtering of candidate lncRNAs. **A** Differentially expressed lncRNAs between tumour and normal tissues. The red point stood for the upregulated lncRNAs and the blue for downregulations. The lncRNAs without significance were marked with black. **B** The lncRNAs that significantly associated with prognosis after secondary filtering. The red point stood for the HR of corresponding lncRNAs higher than 1 and the blue point for HR less than 1. **C** The lncRNAs satisfied the requirements both differentially expressed and prognostic. Overlapping genes in (**C**) were labeled in (**A**) and (**B**). FDR: false discovery rate; FC: fold change; HR: hazard ratio; CI: confidence interval

The expressions profiles of a total of 13,413 lncRNAs and their corresponding genes were downloaded from the training data sets; 2,578 of these lncRNAs were differentially expressed between normal and tumour tissues (Fig. 1A). A list of 944 metabolism-related genes was obtained, and we screened 964 metabolism-related lncRNAs that met the criteria. Subsequently, univariate cox regression analysis of metabolism-related lncRNAs was performed to further mine the potential lncRNAs, and we found that 23 lncRNAs were significantly associated with CRC patient OS (Fig. 1B). Ten differentially expressed metabolism-related lncRNAs with prognostic value were preserved as candidates for the following study (Fig. 1C).

### Construction of the prognostic risk signature

After we obtained the candidate prognosis-related metabolic lncRNAs, we performed LASSO regression to build the prognostic signature and determine the coefficients. Finally, 8 lncRNAs were enrolled in the signature, and each coefficient represented the weight of the expression of the corresponding lncRNA. The risk score for each CRC patient was calculated by formula considering the expression status of the included metabolism-related lncRNAs and their corresponding coefficients (P < 0.05, Table 1).

**Table 1:** Prediction signature for survival

lncRNA	Coefficient
DICER1-AS1 (Ma et al. 2020a)	0.238729835
PCAT6 (Wu et al. 2019)	0.170119979
GAS5 (Cheng et al. 2019; Ni et al. 2019)	0.004528501
PRR7-AS1	0.397473407
MCM3AP-AS1 (Ma et al. 2020b)	0.174037077
GAS6-AS1	0.008075174
LINC01082 (Xiong et al. 2019; Huang et al. 2019)	-0.173104811
ADIRF-AS1	0.025255372

### Evaluation of the prognostic signature containing metabolism-related lncRNAs

**Fig. 2:**
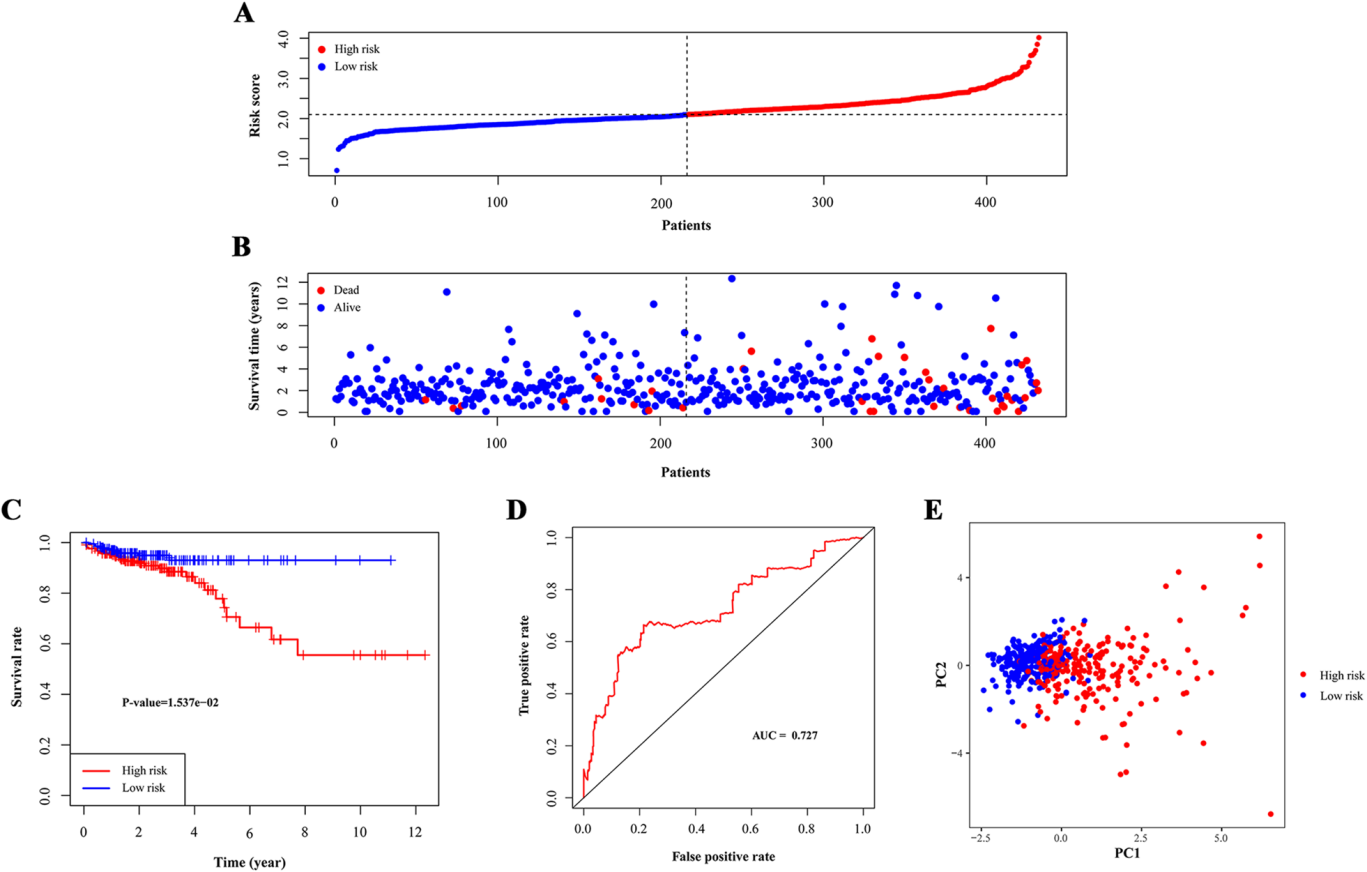
Test of signature in training cohort. **A** Distribution of risk scores in high-risk group and low-risk group. Red point indicated CRC patient in high-risk group and blue indicated low-risk. **B** Distribution of survival status of CRC patients in high-risk group and low-risk group. Blue point represented alive and red point for death. **C** Survival curve of OS. Red line depicted the survival of high-risk patients and blue line for low-risk patients. **D** ROC Curve for risk score. **E** Risk stratification visualized by PCA. AUC: area under curve. PC: principal component

**Fig. 3:**
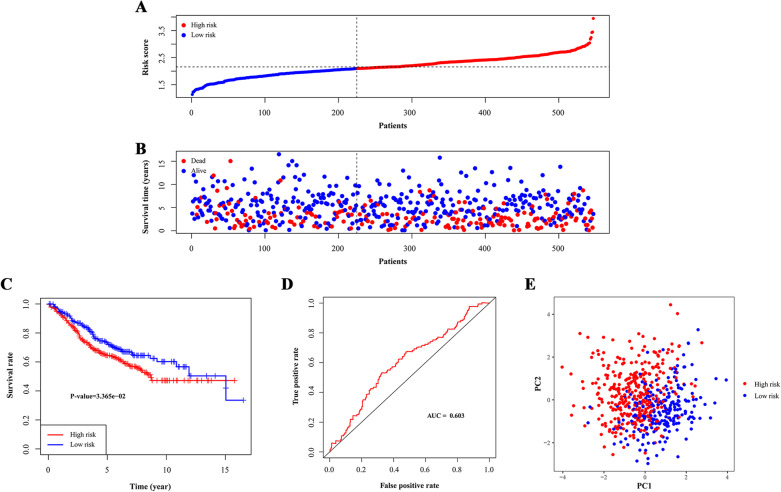
Test of signature in validation cohort. **A** Distribution of risk scores in high-risk group and low-risk group. Red point indicated CRC patient in high-risk group and blue indicated low-risk. **B** Distribution of survival status of CRC patients in high-risk group and low-risk group. Blue point represented alive and red point for death. **C** Survival curve of OS. Red line depicted the survival of high-risk patients and blue line for low-risk patients. **D** ROC Curve for risk score. **E** Risk stratification visualized by PCA. AUC: area under curve. PC: principal component

The risk scores of CRC patients from the TCGA cohort were calculated for internal assessment, and the GEO cohort was used for external confirmation. We grouped the training cohort and the validation cohort into high- and low-risk groups according to the median score of the training cohort (Figs. 2A and 3A). The high-risk groups of CRC patients had higher mortality rates than the low-risk groups in both the training cohort (27/216 versus 10/216) and the validation cohort (117/322 versus 67/225) (Figs. 2B and 3B). High-risk patients had a lower five-year OS rate than low-risk patients in both cohorts (P < 0.05, Figs. 2C and 3C). In the validation cohort, high-risk survival was higher than the low-risk group in the fifteen-year time-span because we calculated the OS, and this prediction would not be tumour type-specific with confounding factors over an increasing number of years. The confounding factors might be treatment effect or physical illnesses such as cardiovascular diseases which worsen with years. The survival analysis of RFS in training cohort also showed better prognosis for low-risk patients (P < 0.05, Additional file 1: Figure S1).

Additionally, the area under the ROC curve (AUC) values for three-year survival in the training and validation cohorts were 0.727 and 0.603, which indicated that the signature had good predictive efficacy (Figs. 2D and 3D).

Then, we performed PCA to assess the distinct distribution between the high- and low-risk groups. Patients tended to separate into two clusters, which clearly indicated that the status of CRC patients in the two risk score groups was different (Figs. 2E and 3E).

### Analysis of the correlation of the metabolism-related lncRNA prognosis signature with clinical features

We then analysed the correlation between the risk scores from the metabolism-related lncRNA prognosis signature and the clinical parameters of the CRC patients from the training cohort. There were no significant differences between risk groups in terms of age, sex and TNM stage (P > 0.05, Additional file 1: Figure S2).

### Construction of the co-expression network and functional enrichment analysis

**Fig. 4:**
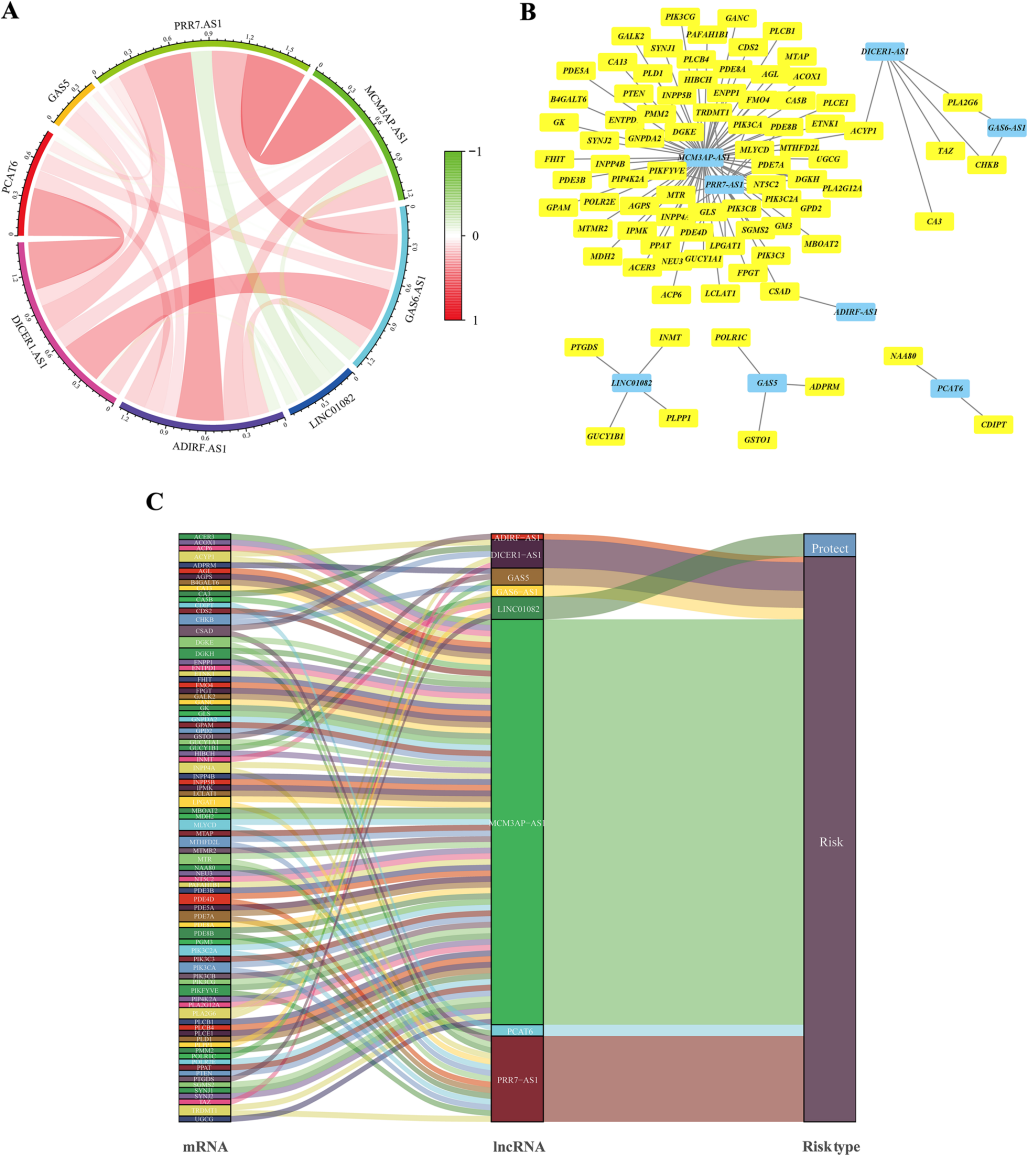
Expression analysis of the metabolism-related lncRNAs prognostic signature according to co-expressed lncRNA-mRNA. **A** Co-expression analysis of lncRNAs with coefficients annotated. **B** The lncRNA-mRNA co-expression regulatory network based on the metabolism-related lncRNAs and highly relevant genes. **C** A Sankey diagram was used to visualize the co-occurrences of mRNAs, lncRNAs and risk types

As shown in Fig. 4A, the lncRNAs in the prognostic signature were closely correlated, which reflected integral consistency. Considering the direct regulation between lncRNAs and mRNAs in the initiation and progression of CRC, a co-expression network was constructed. The lncRNA-mRNA co-expression network contained 103 lncRNA-mRNA pairs that met the threshold, and 85 mRNAs were significantly correlated with the lncRNAs in our prognostic signature (Fig. 4B). MCM3AP-AS1 and PRR7-AS1 might be the major components and are also shown in the Sankey diagram (Fig. 4C). Notably, LINC01082 was the only protective factor among the included lncRNAs.

**Fig. 5:**
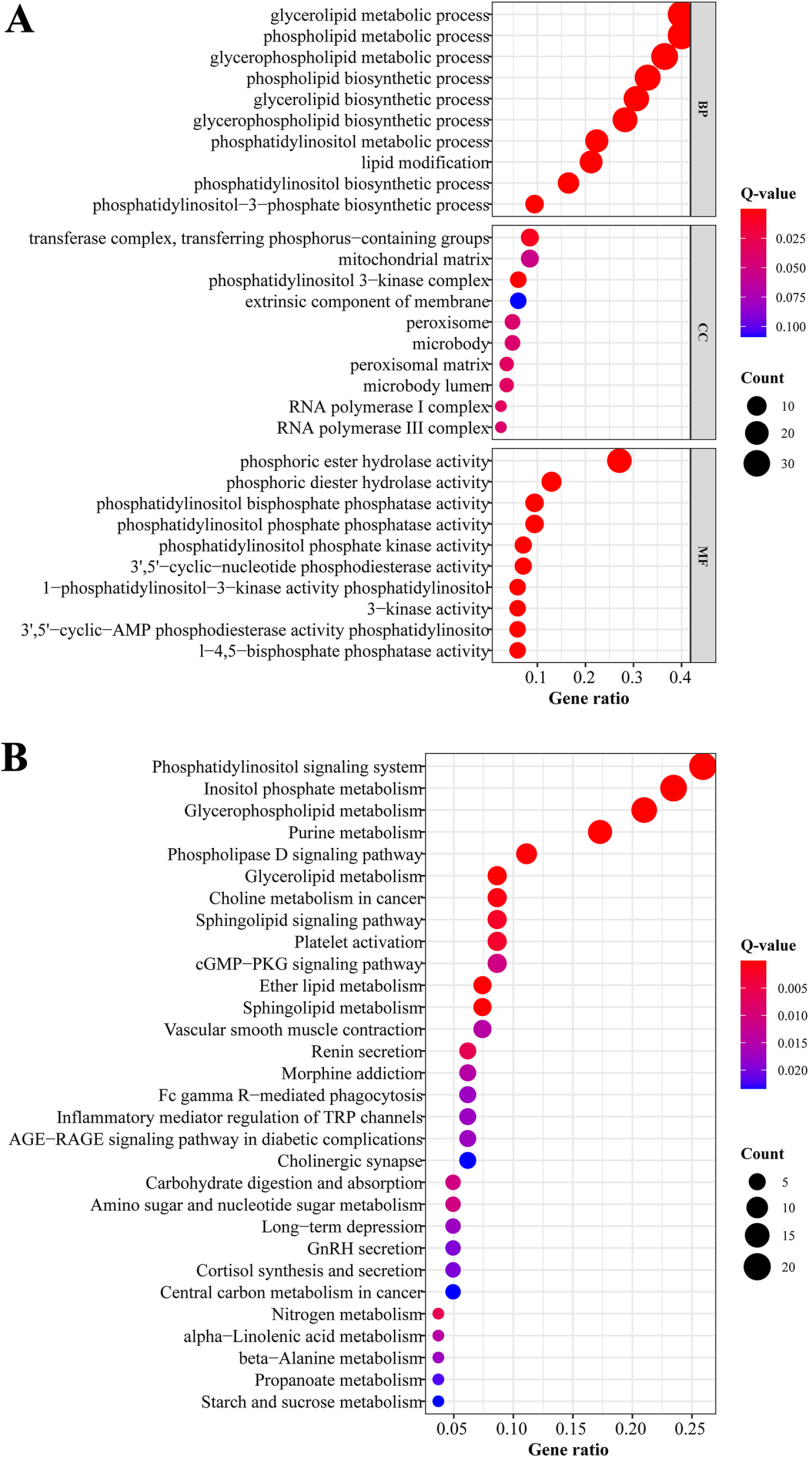
Functional analysis of the mRNAs co-expressed with included lncRNAs. **A** GO analysis of highly related mRNAs. **B** KEGG analysis of highly related mRNAs. BP: biological process. CC: cellular component. MF: molecular function

We performed GO analysis of the mRNAs co-expressed with the 8 lncRNAs, and the top three GO terms for biological processes were the glycerolipid metabolic process, phospholipid metabolic process and glycerophospholipid metabolic process (Fig. 5A). The majority of the enriched KEGG pathways were related to metabolic functions, as expected, and the top three significantly enriched pathways involved the phosphatidylinositol signalling system, inositol phosphate metabolism and glycerophospholipid metabolism (Fig. 5B).

### Analysis of immune status between low- and high-risk groups

**Fig. 6:**
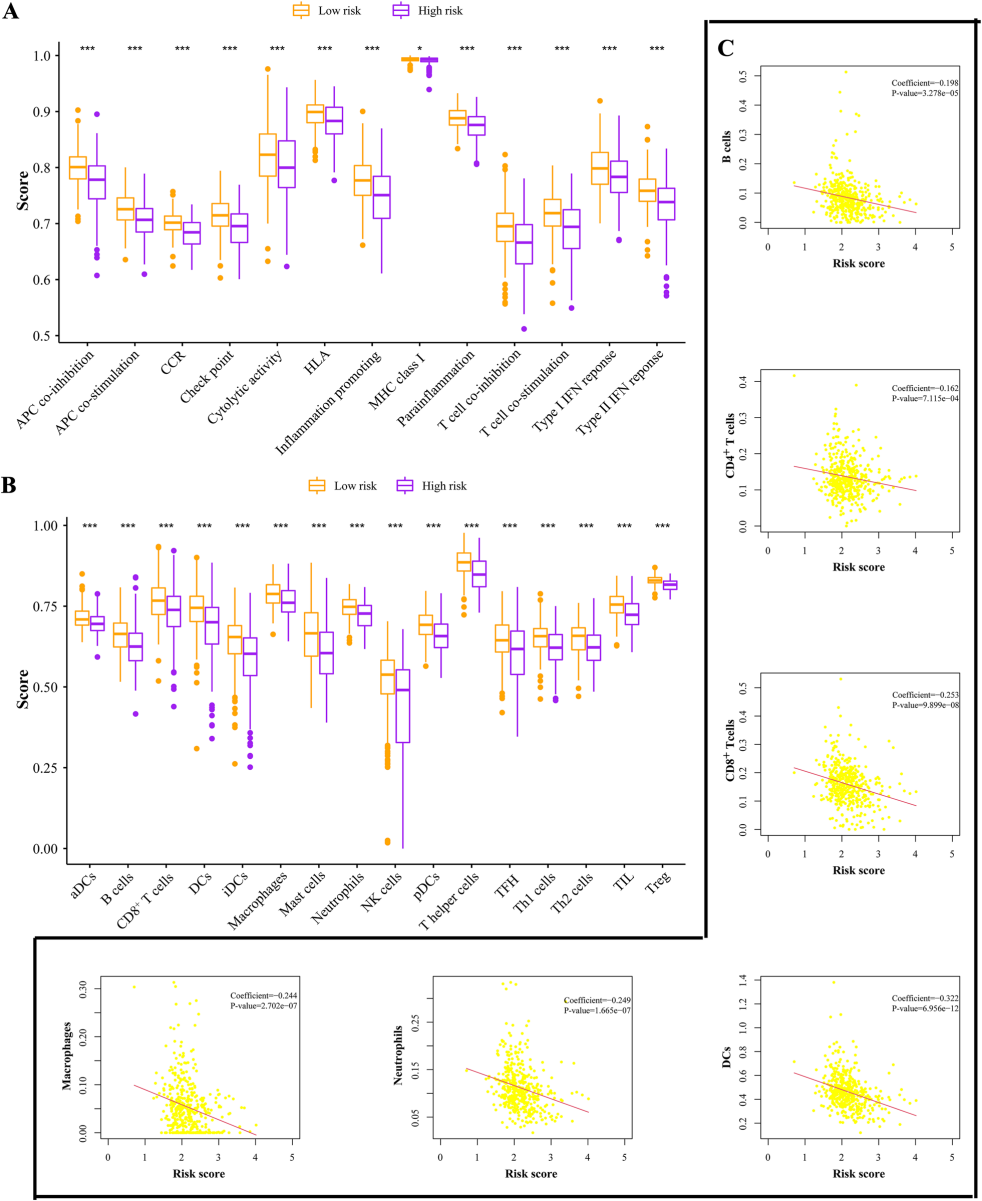
Immune features in the signature. **A** Comparisons of immune functions in different risk groups. **B** The infiltration fractions of immune cells in different risk groups. **C** Estimation of the coefficients for risk score with B cells, CD4^+^ T cells, CD8^+^ T cells, DCs, neutrophils and macrophages. *P < 0.05, **P < 0.01, ***P < 0.001

Our work focused primarily on the metabolic features of CRC patients, but we still explored the immune characteristics of the signature subgroups by assessing the cells in the microenvironment. Interestingly, our ssGSEA results revealed that immune functions such as cytolytic activity, IFN response and inflammation promotion were all significantly increased in the low-risk subgroup (Fig. 6A). The infiltration fractions of CD4^+^ T cells, CD8^+^ T cells, B cells, neutrophils, dendritic cells and macrophages were also higher in the low-risk group in accordance with the infiltration data from TIMER (Fig. 6B–C). Our investigation indicated that the low-risk group had elevated immune activity, which might contribute to antitumour effects.

### Evaluation of the prognostic value of the risk score and construction of a nomogram to predict survival

**Fig. 7:**
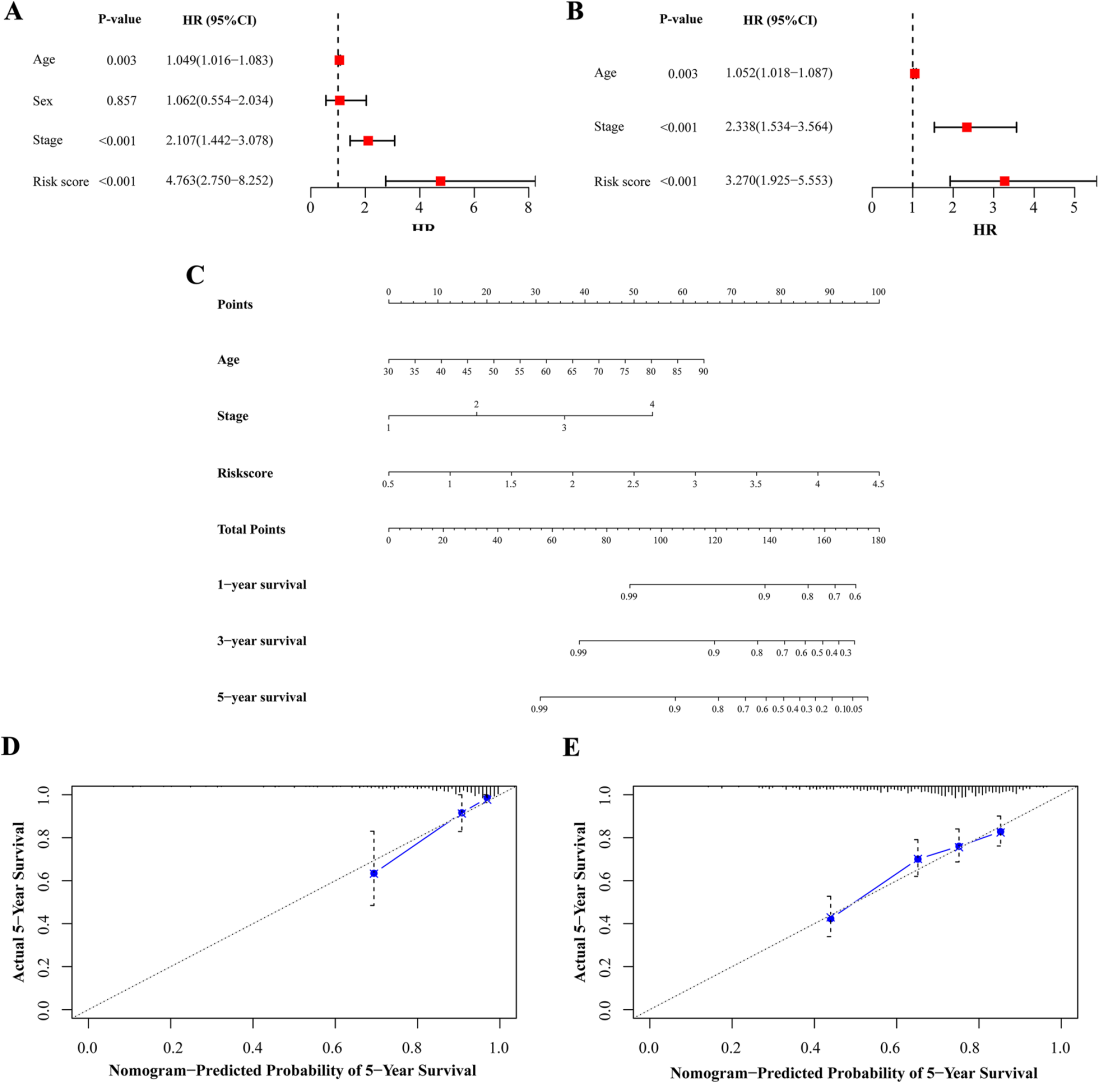
Assessing risk factors and constructing nomogram of prognosis. Univariate analysis (**A**) and multivariate analysis (**B**) were performed for screening of risk factors. **C** The predicted one-, three-, five-year survival rates of CRC patients based on the prognostic nomogram constructed using the risk score from metabolism-related lncRNA prognostic signature and clinicopathological parameters. Calibration curves showed the concordances between predicted and observed five-year survival rates of CRC patients based on the nomogram after bias corrections in training cohort (**D**) and validation cohort (**E**). HR: hazard ratio; CI: confidence interval

We pooled the metabolism-related lncRNA prognostic signature and clinical parameters (age, sex and TNM stage) from the univariate analysis of the training cohort to evaluate the value of the risk score for predicting prognosis. The results showed that the age, stage and risk score, but not sex, of CRC patients were correlated with prognosis (P < 0.05, Fig. 7A). The multivariate analysis indicated that age, stage and risk score might be independent predictive factors for patients, and the HR of the prognostic signature was higher than that of stage (P < 0.05, Fig. 7B).

Nomograms are frequently used to predict patient survival based on the score reflecting the values of several prognostic variables (Balachandran et al. 2015). We also constructed a nomogram to estimate the probability of survival at one, three and 5 years. The predictive factors identified from the multivariate analysis, including age, stage and the metabolism-related lncRNA prognostic signature, were used to construct the nomogram for OS (Fig. 7C). The C-index value of the nomogram was 0.806. The calibration curves depicting the actual and nomogram-predicted survival of the training and validation cohorts at five years were relatively in accord with the reference lines (Fig. 7D–E). These results suggest that the nomogram including our prognostic signature is precise and reliable.

## Discussion

As non-protein-coding transcripts, lncRNAs are typically not translated into proteins and actually exert their functions by regulating proteins and RNA molecules or other transcriptional processes (Ulitsky et al. 2011). These non-coding transcriptome components activate specific mechanisms in the processes of molecular and cellular biology. In addition to regulating gene expression, lncRNAs can also regulate interacting proteins and RNAs. The main effects of lncRNAs on biological behaviour could be completely independent of the encoded RNAs or their production, and in-depth study is needed (Anderson et al. 2015; Kopp and Mendell 2018). LncRNAs are aberrantly expressed in various tumours and can be stably detected in specific cancers (Bhan et al. 2017). Their ability to indicate disease severity in malignant diseases in a non-invasive manner makes them attractive and suitable candidates as preventive and therapeutic targets, especially in personalized treatment (Vitiello et al. 2015). A previous study focusing on the transcriptome revealed that a number of lncRNAs participate in the regulation of CRC pathogenesis and progression through chromosome modification or other transcriptional processes (Gupta et al. 2010). In addition, the activation of signalling pathways such as Wnt/β-catenin mediated by lncRNAs plays critical roles in CRC genesis (Han et al. 2015; Tuupanen et al. 2009).

Previous evidence confirmed that lncRNAs are tightly associated with the metabolic process in cancer patients (Lin 2020). LncRNAs could influence glycometabolism by regulating the expression of glucose transporters and enzymes or altering metabolism-related signalling pathways (Fan et al. 2017). The abnormal expression of lncRNAs in CRC patients might lead to the dysregulation of key genes in lipid catabolism (Muret et al. 2019). LncRNAs function as mediators of metabolism and are expressed during tumour progression in CRC patients. We could take advantage of these interrelationships to precisely estimate the biological characteristics in CRC and propose potential clinical solutions for patients.

Here, our study used transcriptome data to screen metabolism-related lncRNAs associated with CRC patient prognosis. A signature based on the expression of 8 metabolism-related lncRNAs was constructed, and we assessed the credibility of the signature with internal and external cohorts. The risk stratification shown in our study was verified in multiple ways, and a nomogram for survival prediction was built for clinical application. Almost all lncRNAs included in the signature were previously confirmed to be associated with CRC according to external studies. DICER1-AS1 and LINC01082 modulate the proliferation, migration and invasion of CRC cells via different biological mechanisms (Ma et al. 2020a; Xiong et al. 2019). LINC01082 has been confirmed as an optimal diagnostic lncRNA biomarker for CRC patients by bioinformatics and polymerase chain reaction (PCR) in previous study (Huang et al. 2019). Low expression of PCAT6 attenuates the chemoresistance of CRC to 5-fluorouracil (Wu et al. 2019). GAS5 was correlated with a better prognosis and involved as an important node in CRC competing endogenous RNA (ceRNA) network (Cheng et al. 2019). N6-methyladenosine-modified GAS5 could regulate the activation of YAP signaling and inhibit CRC progression (Ni et al. 2019). In CRC tissues and cells, MCM3AP-AS1 was confirmed to regulate cell cycle progression by influencing G1 arrest (Ma et al. 2020b). PRR7-AS1 and ADIRF-AS1 in the signature were newly identified as the prognostic markers in CRC. We focused on the lncRNA associated with metabolic process in tumour progression and found immune-related clues based on data mining which provided novel thought of clinical application comparing to previous studies (Mu et al. 2020; Qin et al. 2021).

The signature presented in our study might still have some limitations that potentially limit its practicality and may need more improvements. First, we performed research based on transcriptome data from public databases with external sets for validation, but more samples and clinical trials are needed to confirm its effectiveness in large populations. Second, fundamental experiments and deep investigations of the possible mechanisms of the metabolism-related lncRNAs in our study are needed support the rationale for utilizing the signature.

## Conclusions

We constructed a signature based on the expression status of metabolism-related lncRNAs in CRC patients with different methods of validation. A risk signature was constructed and incorporated into a predictive nomogram for clinical application. This study provides a useful tool for early diagnosis and prognosis evaluation for CRC.

## Supplementary Information

**Additional file 1: Table S1.** Clinicopathological characteristics of CRC patients. **Figure S1.** Survival curve of RFS in training cohort. **Figure S2.** Correlation between the risk score in the signature and clinical variables.

## Data Availability

The datasets generated and/or analysed during the current study are available from the corresponding author on reasonable request.

## Abbreviations

CRC: Colorectal cancer
lncRNA: Long non-coding RNA
TCGA: The Cancer Genome Atlas
GEO: Gene Expression Omnibus
KEGG: Kyoto Encyclopedia of Genes and Genomes
ROC: Receiver operating characteristic
ssGSEA: Single sample Gene Set Enrichment Analysis
AUC: Area under curve
CEA: Carcinoembryonic antigen
OS: Overall survival
TNM: Tumour-node-metastasis
FC: Fold change
FDR: False discovery rate
HR: Hazard ratio
PCA: Principal component analysis
GO: Gene ontology
APC: Antigen presenting cell
CCR: Chemokine receptor
HLA: Human lymphocyte histocompatibility antigen
MHC: Major histocompatibility complex
IFN: Interferon
DC: Dendritic cell
NK: Natural killer
Th: T helper
TFH: T follicular helper
TIL: Tumour-infiltrating lymphocyte
Treg: Regulatory T cell
TIMER: Tumour Immune Estimation Resource
C-index: Concordance index
LASSO: Least absolute shrinkage and selection operator
RFS: Relapse free survival
CI: Confidence interval
PCR: Polymerase chain reaction
ceRNA: Competing endogenous RNA
