# Supplementary material for: Long non-coding RNA profile study identifies a metabolism-related signature for colorectal cancer
Source: Mol Med. 2021 Aug 3;27:83. doi: 10.1186/s10020-021-00343-x (PMC8336290; doi:10.1186/s10020-021-00343-x)
Supplement: Supplementary file 1 — Additional file 1: Table S1. Clinicopathological characteristics of CRC patients. Figure S1. Survival curve of RFS in training cohort. Figure S2. Correlation between the risk score in the signature and clinical variables. [file 10020_2021_343_MOESM1_ESM.docx]

**Table S1. Clinicopathological characteristics of CRC patients**

| Characteristics | Training cohort (n=432) | Validation cohort (n=547) |
| --- | --- | --- |
| Sex, n |  |  |
| Male | 235 | 301 |
| Female | 197 | 246 |
| Age, y |  |  |
| Median | 66 | 68 |
| Range | 31 to 89 | 22 to 97 |
| Stage, n |  |  |
| Ⅰ | 89 | 31 |
| Ⅱ | 164 | 255 |
| Ⅲ | 130 | 202 |
| Ⅳ | 49 | 59 |


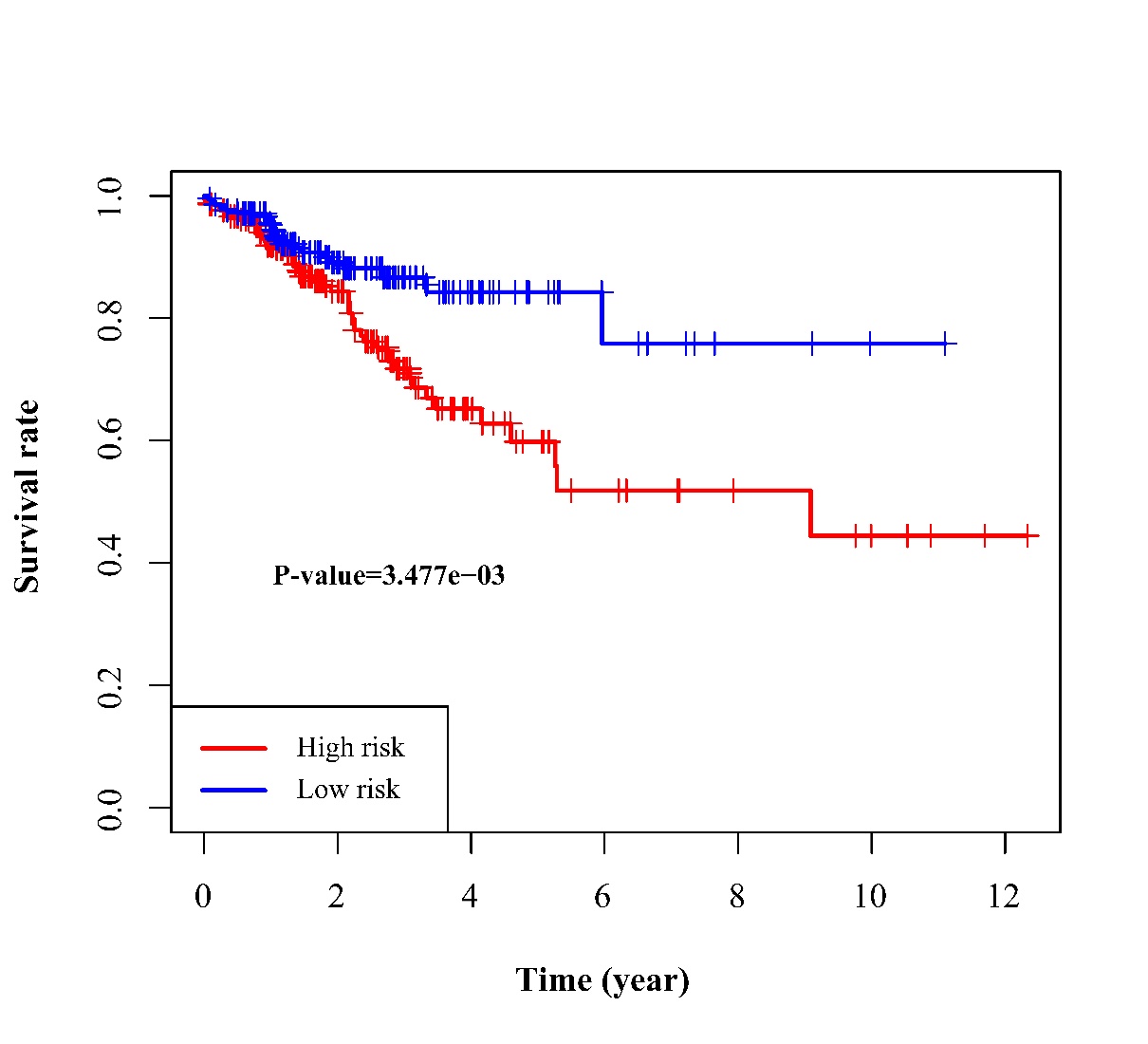


**Figure S1.** **Survival curve of RFS in training cohort.**

Red line depicted the survival of high-risk patients and blue line for low-risk patients.


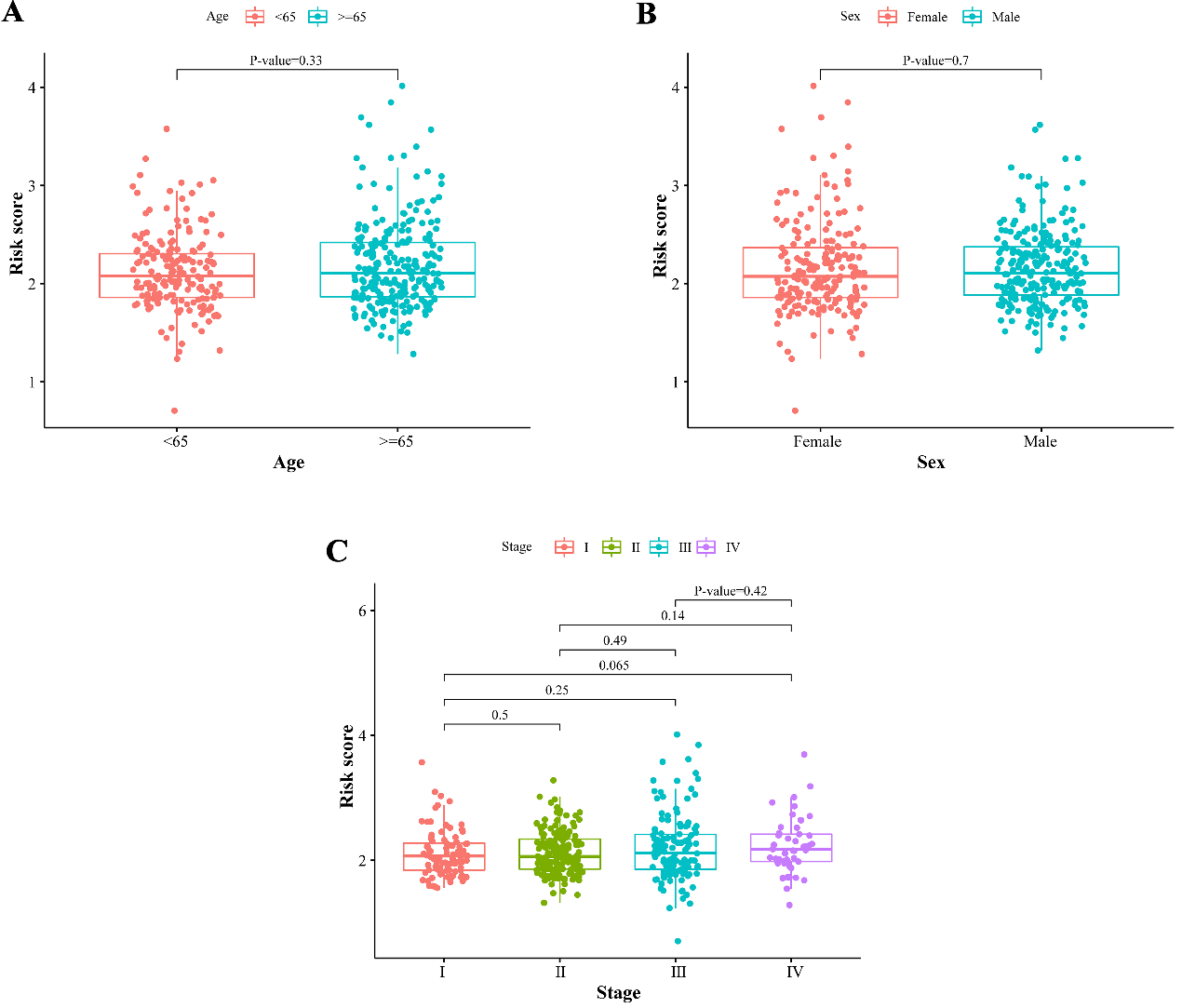


**Figure S2. Correlation between the risk score in the signature and clinical variables.**

(A–C) represent age, sex, TNM stage respectively.
